# Characteristics of the lung microbiota in lower respiratory tract infections with and without history of pneumonia

**DOI:** 10.1080/21655979.2021.1997563

**Published:** 2021-12-11

**Authors:** Lingling Hong, Yuqing Chen, Ling Ye

**Affiliations:** Department of Respiratory Critical Care Medicine, The Fifth Hospital of Xiamen, Xiamen, Fujian Province, China

**Keywords:** Lower respiratory tract infections, history of pneumonia, lung microbiota, diagnosis

## Abstract

Lung microbiota plays an important role in many diseases including lower respiratory tract infections (LRTI) and pneumonia. This study aimed to explore the effects of community-acquired pneumonia (CAP) on microbial diversity and identify potential biomarkers of respiratory tract in CAP LRTI patients. In the current study, a comprehensive bioinformatics analysis was performed based on metagenomic next-generation sequencing technology, followed by alpha and beta diversity, LEfSe, and co-occurrence network analysis, and random forest model construction. Our results showed that CAP dramatically influenced taxon abundance, and the significant differences in microbiota including *Proteobacteria, Bacteroidetes, Euryarchaeota, Firmicutes* and *Spirochetes* were observed at the phylum level. Co-occurrence network selected out novel modules involved in microbial proliferation-associated pathways. A random forest model screened *Klebsiella pneumoniae* and *Bacillus cereus* as potential diagnostic biomarkers with high AUC values. The microbial composition was different between CAP LRTI patients and non-CAP LRTI patients. *Klebsiella pneumoniae* and *Bacillus cereus* were strongly associated with increased severity of LRTI with a pneumonia history. Our findings provided an insight for a better understanding of community and structure of lung microbiota for future diagnosis and treatment in LRTI patients with a history of pneumonia. Moreover, these microbes were considered as potential biomarkers for predicting the risks for the treatment strategies of LRTI.

## Introduction

Lower respiratory tract infection (LRTI) is the most common syndrome leading to death in the world. LRTI may also be caused by noninfectious syndromes [1]. Previous studies have shown that potential pathogens can cause a variety of diseases, including LRTI and pneumonia [2]. For example, previous evidence suggests that *Staphylococcus aureus* is associated with the course of LRTI [3]. Community-acquired pneumonia (CAP) is often misdiagnosed and inappropriately treated as a common infection [4]. It has been showed more than 2 million children die from pneumonia each year worldwide [5]. Additionally, *Mycoplasma Pneumoniae* is considered to be a common causative agent of CAP [2], and acute LRTI [6]. Antibiotics commonly used to treat *Mycoplasma Pneumoniae* are still necessary for LRTI secondary to *Mycoplasma Pneumoniae* in children [6]. *Klebsiella pneumonia* has been directly found in LRTI patients, and it was identified as a etiological factor causing LRTI based on clinical features [7,8]. Nowadays, it is still a challenge for clinicians and microbiologists to reduce pulmonary complication and eliminate toxicity of empirical antibiotic therapies. By understanding the differences in the microbiological composition of patients with CAP and non-CAP LRTI, the disease risk can be predicted and treatment plans can be developed accordingly [9].

Shotgun and targeted metagenomics based on next-generation sequencing (NGS) technology showed that the dysbiosis of respiratory tract microbiome plays important roles in the pathological process of pneumonia [10,11]. NGS-mediated metagenomics analysis provide a new insight into composition and function of microbiome in physiological and pathological processes [12]. Results from NGS data have revealed *Haemophilus influenzae* as an important pathogen in chronic obstructive pulmonary disease (COPD) exacerbations [13]. In addition, *Acinetobacter, Bacillus* and *Staphylococcus* were recognized as pathogenic bacteria associated with LRTI [14]. The balance of microbial composition is affected by inflammatory responses and antibiotic application [15]. Moreover, acute and chronic lung diseases change the migration and elimination of lung microbiome [16]. It has been demonstrated changes in the dynamics of the respiratory microbiota are associated with pneumonia, and pneumonia causes the lung microbiome out of balance [12]. The history of pneumonia was screened as a strong risk factor for COPD exacerbation [17]. The hospitalized patients with a history of pneumonia showed a higher risk of developing lung disease, compared to non-CAP patients [18]. However, the dynamics and mechanisms of respiratory microbiota among patients with CAP LRTI or non-CAP LRTI remain obscure.

Here, we expected that CAP LRTI patients display different microbial compositions compared with non-CAP LRTI patients. Hence, to confirm this hypothesis, we aimed to evaluate the changes in the microbiota of bronchoalveolar lavage fluid (BALF) between CAP LRTI patients and non-CAP LRTI patients using metagenomic NGS technology. Meanwhile, a comprehensive and innovative bioinformatics analysis was performed to explore microbiota profiles in CAP LRTI patients and non-CAP LRTI patients. The discriminant model established based on clinical features was used to predict the pathogens of CAP LRTI patients and non-CAP LRTI patients, which provides the basis for detailed bacteriological diagnosis and antibiotics therapy.

## Materials and methods

### Patients and BALF Collection

A total of 6 LRTI patients with a diagnosis of CAP and 8 non-CAP LRTI patients were enrolled during January 132,019 to April 3 2020 from the department of respiratory medicine of the first affiliated hospital of Xiamen University (Fujian, China). The diagnosis of CAP is according to a combination of clinical features (cough, dyspnea, sputum production, pleuritic pain, fever or chills, and malaise) and imaging of the lung by chest radiography. Evaluation of specific pathogens (bacteria, fungi, virus, and mycobacteria) was performed. Non-pneumonia LRTI patients were included with the presence of hyperinflation or new or increased peribronchial infiltrates without alveolar infiltrates on chest radiograph. Patients with at least one day of follow-up were included in the study. Patients were excluded if they had been hospitalized within the previous week, or diagnosed other than an immunocompromised condition, and if no interpretable chest X-ray was obtained. All patients signed the informed consents. The clinical features of patients are presented in Table 1.

**Table 1.** Age and gender of patients with CAP and non-CAP LRTI

	CAP (n = 6)	non-CAP LRTI (n = 8)
**Gender**		
Males, n (%)	6 (100%)	4 (50%)
Females, n (%)	0 (%)	4 (50%)
**Age**		
Mean ± SD	47.83 ± 22.66	47.88 ± 16.81
Range, year	25–75	26–70
> 65 years, n (%)	2 (33.3)	2 (25)

CAP, community acquired pneumonia; LRTI, lower respiratory track infection; SD, standard deviation.

Bronchoalveolar lavage procedure was carried out with reference to a standard safety protocol [19]. BALF samples were collected from LRTI patients with and without a history of pneumonia. BALF sampling was carried out in the right middle lobe or left upper lobe of the lung allograft. After the bronchoscope reaches a wedge position, 50 mL of normal saline were instilled. The wedged position was maintained and normal saline was suctioned. The samples were collected into a separate container, and transferred into a sterile sputum container. After recording the volume, the samples were stored at −20°C.

### DNA extraction and NGS analysis

The collected BALF samples were used for sequencing and bioinformatics analysis. DNA was extracted and purified using QIAamp DNA Microbiome Kit (Qiagen, Hiden, Germany). The microbial DNA was used to generate sequencing library with NEBNext Ultra II DNA library Pre Kit (Illumina, San Diego, CA, USA). The library was purified and its quality was evaluated using agarose gel electrophoresis. After dilution, mixture, denaturation, and re-dilution, the sample was spiked with PhiX according to the MiniSeq System Denature and Dilute Libraries Guide. Following the MiniSeq Local Run Manager Software Guide, the sequencing was performed on an Illumina MiniSeq system with the high output reagent kit 150 cycles. CapitalBio Corporation (Beijing, China) was commissioned to perform the DNA extraction and sequencing.

### Bioinformatics analysis

The raw data were subjected to high-quality filtration and trimmed through CLC genomics workbench software (Qiagen). The filtered reads were assembled into contigs and mapped back to the contigs for calculating numbers of reads per contigs. Bowtie2 (https://github.com/topics/bowtie2) was used to select human-derived sequences from metagenomic data and aligned with the human genome in NCBI database (GRCh38). The obtained quality-filtered reads were run through the UPARSE (http://www.drive5.com/uparse) and subject to operational taxonomy unit (OTU) clustering with a similarity threshold of 98%. All assigned reads were subjected to downstream analysis, including alpha and beta diversity assessments using the Quantitative Insights into Microbial Ecology (QIIME) package in R software.

To explore significant differences between CAP LRTI and non-CAP LRTI patients, the linear discriminant analysis effect size (LEfSe) analysis was carried out to identify specific OTUs. To select out the diagnostic factors, the receiver operating characteristic (ROC) curves were illuminated using the pROC R package. The area under the ROC curve (AUC) value was used to screen diagnostic genes with AUC > 0.7 as the threshold. To explore the functional enrichment analysis of novel genes, the GO and Kyoto Encyclopedia of Gene and Genomes (KEGG) analysis were performed using ‘clusterProfiler’ R package.

### Statistical analysis

All statistical analyses were performed using R software. Wilcoxon signed rank test was used to compare alpha diversity measures. Nonmetric multidimensional scaling was used to compare beta diversity measures. The correlation between two groups was analyzed, and the significance difference was accepted with Spearman correlation coefficient > 0.6 and Benjamini Hochberg *P*-value < 0.05 as the cutoff criteria.

## Results

The homeostasis of lower respiratory tract microbiome maintains the balance of immune system, while its dysbiosis triggers lung inflammation [14]. Here, we speculated that LRTI patients hospitalized for CAP present a different microbial composition from non-CAP LRTI patients. Thus, to prove that CAP shows effects on microbiome in LRTI patients, we assessed the changes of microbiome in BALF between CAP LRTI and non-CAP LRTI patients.

### Effects of CAP on microbial community in BALF samples from patients with LRTI

**Figure 1.**
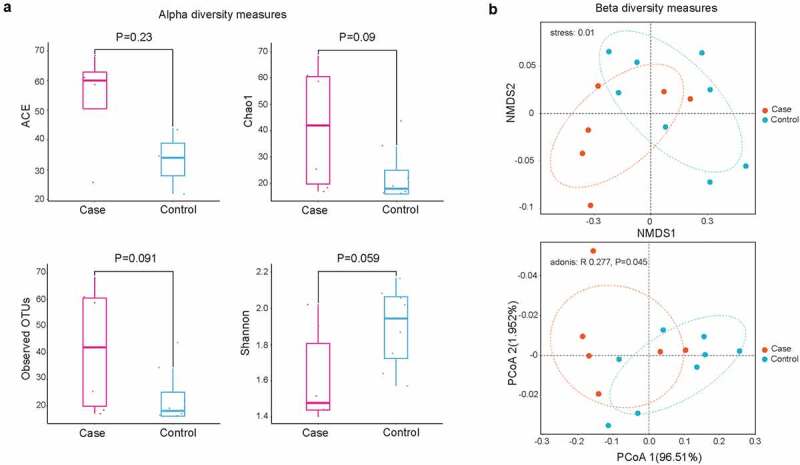
Diversity analysis for microbiota in bronchoalveolar lavage fluid. (a) Alpha diversity analysis showed significant differences in abundance-based coverage estimator (ACE), taxonomy-based richness (Chao1), observed OTUs, and Shannon index; (b) Beta diversity analysis by non-metric multidimensional scaling (NMDS, up panel) and principal co-ordinates analysis (PCoA, down panel)) suggesting the community structure of microbiota

To investigate the role of CAP in lung microbiota composition, NGS technology was used to produce clean reads from 14 samples. In our results, a total of 300 OTUs were commonly detected in two groups, while 8 OTUs were particularly found in non-CAP LRTI patients and 6 OTUs were uniquely detected in CAP LRTI patients. There was no significant difference in ACE (*P* = 0.23), chao 1 (*P* = 0.09), observed OTUs (*P* = 0.091), and Shannon index (*P* = 0.059) between case group (CAP LRTI patients) and control group (non-CAP LRTI patients) (Figure 1(a)), suggesting that the alpha diversity of microbiome was relatively consistent between case group and control group. PCoA analysis revealed that the weighted UniFrac distance was dramatically different between case group and control group (*P* = 0.045, Figure 1(b)). These results indicated that CAP shows effects on microbial community in BALF samples from patients within LRTI.

### Effects of history pneumonia on the abundance of microbial taxon in LRTI patients

**Figure 2.**
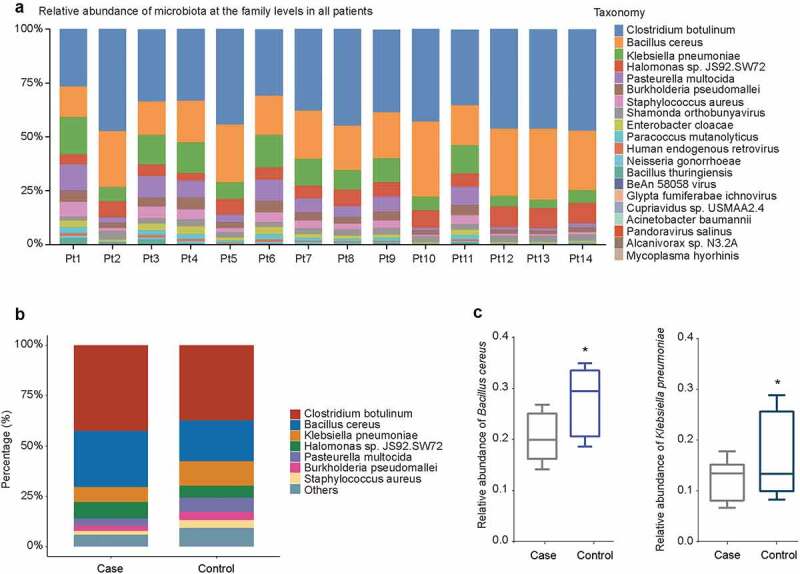
Relative abundance of microbiota at the genus level. (a) Relative abundance of microbiota in individual patients with CAP LRTI patients (Pt9, Pt10, Pt11, Pt12, Pt13, and Pt14) and non-CAP LRTI patients (Pt1, Pt2, Pt3, Pt4, Pt5, Pt6, Pt7, and Pt8). (b) Relative abundance of microbiota in case group (CAP LRTI patients) and control group (non-CAP LRTI patients). (c) The relative abundance of *Bacillus cereus* and *Klebsiella pneumonia* with the most significant difference between case group (CAP LRTI patients) and control group (non-CAP LRTI patients). Student *t*-test was used to evaluate significant differences between the groups, *P < 0.05

To explore the effect of history of pneumonia on the abundance of microbiome with different taxonomy, the beta diversity was analyzed. At the family level, *Clostridium botulinum* showed the highest abundance in 14 patients, followed by *Bacillus cereus* and *Klebsiella pneumoniae* (Figure 2(a)). The taxon abundance of microbiota (*Clostridium botulinum, Bacillus cereus, Klebsiella pneumoniae, Halomonas sp. JS92.SW72, Pasteurella multocida, Burkholderia pseudomallei*, and *Staphylococcus aureus*) were compared between case group and control group, as shown in Figure 2(b). The abundance of *Glypta fumiferanae ichnovirus* was significantly increased in case group compared to control group (*P* = 0.00496), followed by *Candidatus Portiera aleyrodidarum* (*P* = 0.00795) (Table S1). The relative abundance of *Bacillus cereus* and *Klebsiella pneumoniae* was decreased in case group compared with control group (*P* < 0.05, Figure 2(c)).

### LEfSe analysis identifying the dysregulated microbiota in CAP LRTI patients

**Figure 3.**
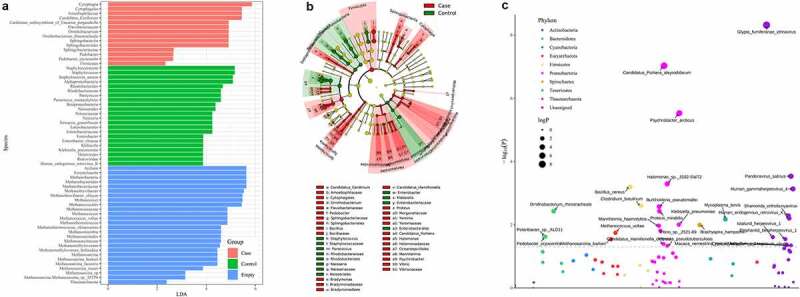
Effects of pneumonia history on microbiota composition in BALF. (a) The linear discriminant analysis (LDA) scores of taxa presented the difference in microbiome composition between CAP LRTI patients and non-CAP LRTI patients; (b) Taxonomic cladogram by LEfSe analysis showed the changes of microbiome in patients with CAP LRTI patients and non-CAP LRTI patients; (c) Manhattan plots showed the OTUs in case group (CAP LRTI patients) and control group (non-CAP LRTI patients). The colors of the dot represent the different taxonomic affiliations of the OTUs (phylum level). The size corresponds to their relative abundance in the respective samples. The dashed line corresponds to the significance threshold of *P*-values (*P* < 0.05)

In order to screen for potential microbial biomarkers that differ in abundance between CAP LRTI patients and non-CAP LRTI patients, LEfSe analysis was performed using the LEfSe R package to discover high-dimensional biomarkers. Results showed that there are 47 taxonomic clades showing significant differences between CAP LRTI patients and non-CAO LRTI patients. Several microbes were detected as potential markers in CAP LRTI patients non-CAP LRTI patients, including *Candidatus, Cytophagia*, and *Spirochetes* (Figure 3(a)). *Cytophagia* showed the highest scores of LDA, suggesting that microbial abundance showed strong effects on the module groups(Figure 3(b)). CAP LRTI patients suggested a significant change in lung microbiota at the phylum level with *P* < 0.05. Our results identified numerous dysregulated microbes including *Proteobacteria, Bacteroidetes, Euryarchaeota* and *Firmicutes* (Figure 3(c)).

### Network analysis revealing a potential interaction consisting of microbiota with different abundance between CAP LRTI and non-CAP LRTI patients

**Figure 4.**
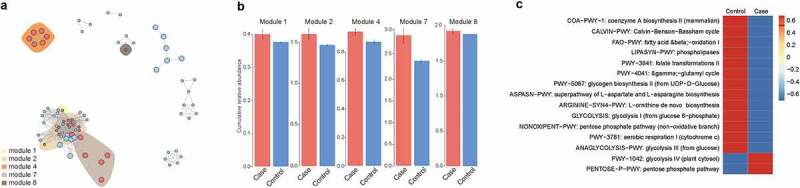
The co-occurrence networks and function analysis of microbiome differently detected in CAP LRTI patients and non-CAP LRTI patients. (a) A strong correlation among module 1, module 2, module 4, module 7, and module 8 shows an effect of CAP on microbial composition of LRTI. (b) The cumulative relative abundance of each module in case group (CAP LRTI patients) and control group (non-CAP LRTI patients). (c) The difference of KEGG pathways is presented between case group (CAP LRTI patients) and control group (non-CAP LRTI patients)

To explore a potential microbial interaction, we selected out novel modules in the co-occurrence network. We subsequently investigated the biological function of microbiota by using Spearman method. A total of 10 modules were identified in the network analysis (Table S2, Figure 4(a)). The modules with significant difference in abundance used for the downstream analysis include module 1, 2, 4, 7 and 8 (Figure 4(b)). These microbials in the differential modules were enriched in microbial proliferation-associated pathways, including glycolysis, pentose phosphate pathway, Calvin-Benson-Bassham cycle and L-ornithine de novo biosynthesis (Figure 4(c)).

### Forest model was used for screening potential pathogenic bacteria for CAP LRTI patients

**Figure 5.**
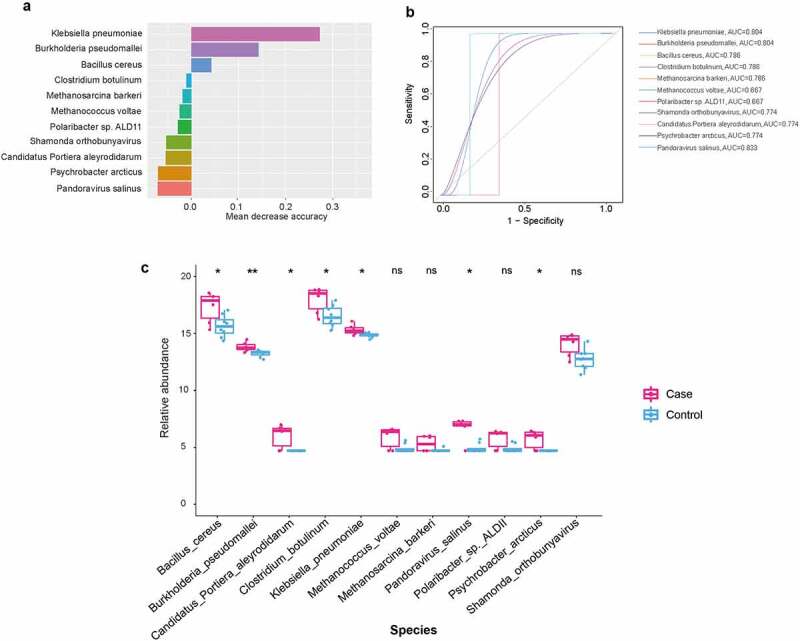
A random forest-based classification model for identifying novel microbiota in CAP LRTI and non-CAP LRTI patients. (a) The OTUs that contributed the most to the model are ranked by mean decrease accuracy. (b) Receiver operating characteristic curve (ROC) of random forest model. (c) The relative abundance of microbiota in random forest models. Student *t*-test was used to evaluate significant difference. *P < 0.05

To explore which microorganisms are influenced by the history of pneumonia, we further applied a random forest model to screen for potentially pathogenic microorganisms. In our results, we also observed high accuracy scores of microbes that were associated with CAP LRTI patients, including *Klebsiella pneumonia, Burkholderia pseudomallei, Bacillus cereus*, and *Methanococcus voltae* (Figure 5a). The receiver operating characteristic (ROC) curves were drawn and AUC values were calculated to evaluate the prediction ability of the random forest model. Our findings demonstrated higher AUC values of the following microbiota, *Klebsiella pneumonia* (AUC = 0.804), *Burkholderia pseudomallei* (AUC = 0.804) and *Bacillus cereus* (AUC = 0.786, Figure 5b). Furthermore, the relative abundance of these microbiota with high accuracy scores was calculated. The results showed an increase in the relative abundance of *Klebsiella pneumonia* (*P* = 0.00992), *Burkholderia pseudomallei* (*P* = 0.00267) and *Bacillus cereus* (*P* = 0.00064) in CAP LRTI patients compared to non-CAP LRTI patients (Figure 5c).

## Discussion

Previous studies showed that the dysregulation of pathogenic microbiota was associated with many complex diseases, including LRTI and CAP [20–22]. Recent studies have shown that lung microbiota were dramatically changed in response to pathological alterations, and patients showed significant differences in community composition during disease progress [16]. Studies demonstrated that the diversity reduction of nasal microbiome including *Rothia* and *Lactobacillus* would increase the risk of pneumonia [23]. The pathogens such as *Mycoplasma pneumoniae* casing pneumonia have been considered as risk factors for asthma [24]. HIV virus increased the risk of LRTI progression [25]. However, the microbiome diversity of CAP LRTI patients remains obscure. Therefore, our study performed the high-throughput sequencing to explore the difference in structure and composition of microbiota community between CAP LRTI patients and non-CAP LRTI patients.

In our present study, the lung microbiota of 6 CAP LRTI patients and eight non-CAP LRTI patients were examined using metagenomic NGS technology. Next, we investigated the diversity and biomarkers for CAP LRTI patients. For alpha diversity indexes, there were no significant difference in the alpha diversity of microbiota in CAP LRTI patients and non-CAP LRTI patients, suggesting that the diversity and richness of bacterial communities were similar between LRTI patients with or without CAP. Our findings conformed to the results of alpha diversity in oral microbiome analyzed by other researches [26]. The microbial composition in lung cancer patients significantly differs between lung cancer patients and healthy controls but not microbial diversity [27]. On the contrary, it has been reported that the outgrowth of pathogens in pneumonia patients led to a decrease in diversity [28]. In the elderly with dental caries, the results of alpha diversity showed more varied communities in healthier ecosystems [29]. In patients with acute respiratory distress syndrome, the alpha diversity is significantly decreased compared to that of control groups [30]. In LRTI patients, the alpha diversity has been reported to decrease compared to that of patients with upper respiratory tract infection [31]. For beta diversity indexes, our results found a dramatical difference in the composition (PcoA, *P* = 0.0045), suggesting a potential effect of pneumonia history on lung microbiota in patients with LRTI. Similarly, studies have demonstrated significant differences in beta diversity of microbiota between the upper and the lower respiratory tract [31]. In upper respiratory tract infection, there is a higher probability in the alteration of lung microbial composition [32]. In saliva microbiota, the results of beta diversity analysis showed that the community structures were dramatically different in *Helicobacter pylori* uninfected and infected patients, showing that pathogenic bacteria affect the community structures of lung microbiota [33]. The results of beta diversity analysis showed a significant difference between patients with or without LRTI [34]. These findings suggested the history of pneumonia influenced the microbial composition of LRTI patients.

Studies reported that the functional abundance of the microbiome depends on the individual and interactive roles of the environment and community structure [35]. The microbiota are involved in the immunological homeostasis in lung mucosa [36]. Furthermore, the microbiota may contribute to the susceptibility to diseases by metabolites-mediated immunological progresses [37]. Our results showed that the dysregulated-microbiota were enriched in metabolites-associated pathways, including pentose phosphate pathway and glycolysis pathway. Previous studies have revealed that SARS-CoV-2 infectivity causes increased abundances of bacterial species associated with glycolysis [38]. The gut bacterial taxa are involved in pentose phosphate in acute leukemia patients, including *Streptococcus, Ruminococcus* and *Veillonella* [39].

The random forest algorithm exhibits a high accuracy and robustness [40]. A recent study showed that a random forest model is used for feature selection and biomarkers screening [41]. The random forest algorithm has been used to identify the gut microbiota as biomarkers in major depressive disorders [42]. Another study showed that a random forest model was established based on oral microbiota, which is used for biomarker selection [43]. To explore the novel microbial markers to distinguish LRTI patients with or without CAP, the random forest analysis was used to screen the biomarkers in the present study. Studies have reported that random forest classification at the genus level was used to select out the gut microbiota as biomarkers for major depressive disorders [42]. In children with juvenile idiopathic arthritis, a random forest model was established to screen 12 genera in gut microbiota as biomarkers using nested cross-validation analysis [44]. In nasopharyngeal microbial composition, the random forest model was used to explore the bacterial genera involving in pneumococcal colonization [45]. In LRTI, the random forest classifier analyses on the bacterial data were performed to discriminate LRTI from healthy individuals [20]. These results showed that a random forest model was considered as a universal method to screen potential novel bacterial genera and biomarkers.

In the present study, the random forest model was constructed to screen the potential novel biomarkers. The obtained results showed that the accumulation in the increasing abundance of *Bacillus cereus* and *Klebsiella pneumoniae* was associated with the lung microbiota profiles of LRTI patients with CAP compared with that in LRTI patients without CAP. Studies showed that microbiome dysbiosis was considered as biomarkers for the development of potential colorectal cancer [46]. In addition, *Bacillus cereus* was regarded as a volatile human pathogen [47]. In pneumonia patients, *Bacillus cereus* contributes to the development of severe pneumonia and it is recognized as a potential pathogen [48]. There were few literatures reporting that *Bacillus cereus* causes LRTI [48,49]. Our results showed *Bacillus cereus* was identified as a marker in LRTI patients with a history of pneumonia. Whether *Bacillus cereus* directly causes the development of LRTI or homeostasis of microbial diversity in pneumonia will be explored in our future study. Interestingly, our findings revealed *Klebsiella pneumonia* as the indicator of CAP LRTI patients using random forest classifier, which got an AUC value of 0.804. Studies have showed that *Klebsiella pneumonia* are the important pathogens causing pneumonia, which are associated with high morbidity and mortality [50,51]. In wheezing episodes in children, *Klebsiella pneumoniae* are predictive markers for the prediction of wheeze [52]. In the diversity of pathogens responsible for LRTI, *Klebsiella pneumonia* were identified as the most predominant single pathogens [53].

Nevertheless, some limitations should be considered in the present study. The metagenomic NGS analysis was performed to examine microbial diversity and identify potential biomarkers, rather than to do some experimental research to explore the mechanisms of *Bacillus cereus* on LRTI progression. Secondly, the biomarkers should be further screened in a broad population.

## Conclusions

This study revealed a difference in microbial composition between CAP LRTI and non-CAP LRTI patients. Further, we identified *Bacillus cereus* as a potential biomarker to predict the risk of LRTI progress, which is conducive to the diagnosis and management of CAP LRTI and non-CAP LRTI patients.

## Data Availability

The datasets used and/or analyzed during the current study are available from the corresponding author on reasonable request.
